# Case Report: Apatinib combined with IMRT concurrent therapy achieves rapid response and significantly prolongs progression-free survival in elderly patients with inoperable locally advanced esophageal squamous cell carcinoma

**DOI:** 10.3389/fonc.2025.1655976

**Published:** 2025-09-15

**Authors:** Juan Liu, Wei Li, Ning Zan, Tingwu Yi, Cheng Li, Shi Liang

**Affiliations:** ^1^ Department of Oncology and Hematology, People’s Hospital of Leshan, Leshan, China; ^2^ Department of Urology, People’s Hospital of Leshan, Leshan, China

**Keywords:** esophageal squamous cell carcinoma, apatinib, intensity-modulated radiation therapy, elderly patients, unresectable, case report

## Abstract

Patients with locally advanced esophageal squamous cell carcinoma (LA-ESCC) typically have a poor prognosis, and concurrent chemoradiotherapy is the primary treatment modality. However, elderly patients often exhibit lower completion rates of concurrent chemoradiotherapy due to multiple comorbidities and reduced treatment tolerance, which directly affects their prognosis. Although apatinib combined with radiotherapy has demonstrated synergistic potential in treating certain solid tumors, its efficacy in elderly patients with LA-ESCC remains unclear. This case report involves an elderly patient with unresectable LA-ESCC who was treated with apatinib combined with intensity-modulated radiation therapy, The patient had comorbidities, including hypertension and diabetes, and declined chemotherapy. We implemented a treatment regimen consisting of intensity-modulated radiation therapy combined with low-dose apatinib. After nine sessions of radiotherapy, the patient’s dysphagia significantly improved, and follow-up computed tomography revealed marked tumor shrinkage. As of July 2024, the patient has achieved a progression-free survival of 71 months without experiencing severe (Grade III/IV) adverse reactions. Therefore, apatinib combined with radiotherapy suggests potential benefits as a concurrent treatment option for elderly patients with unresectable LA-ESCC.

## 1 Introduction

Esophageal cancer (EC) is a highly aggressive and fatal disease, ranking seventh in incidence and sixth in cancer mortality worldwide. Squamous cell carcinoma is the predominant histological type of esophageal carcinoma with its incidence being associated with smoking, alcohol consumption, and malnutrition (1). These factors may also increase the risk of EC in elderly men (2). Surgery is the primary treatment option for early-stage esophageal squamous cell carcinoma. However, by the time most patients seek medical treatment, the disease has already progressed to locally advanced esophageal squamous cell carcinoma (LA-ESCC), making them ineligible for radical resection (3). Patients with unresectable LA-ESCC have poor prognosis and high mortality. Consequently, radiotherapy and chemotherapy have become the most common and essential treatments for esophageal carcinoma (4). However, the local recurrence rate in patients with EC undergoing definitive concurrent chemoradiotherapy (dCRT) is 40–60% (5), with a 5-year overall survival rate of only 10–30% (6). Although dCRT has demonstrated superior efficacy compared with chemotherapy alone, especially in patients with squamous cell carcinoma (7), it can also induce more severe toxic side effects than chemotherapy alone (8). Elderly patients demonstrate poor adherence to combination therapy (9, 10). Elderly patients harbor fears regarding the potential gastrointestinal and skin/mucosal reactions that chemotherapy may cause. Previous studies have confirmed that apatinib combined with radiotherapy is more effective than radiotherapy alone for treating advanced pancreatic cancer and that apatinib has an acceptable safety profile (11). However, there have been no clinical studies on the use of single-agent apatinib in combination with radiotherapy for the treatment of LA-ESCC. This report documents a case in which apatinib combined with intensity-modulated radiation therapy (IMRT) as concurrent therapy resulted in a rapid response and might have clinical relevance in an elderly patient with unresectable LA-ESCC.

## 2 Case description

**Figure 1:**
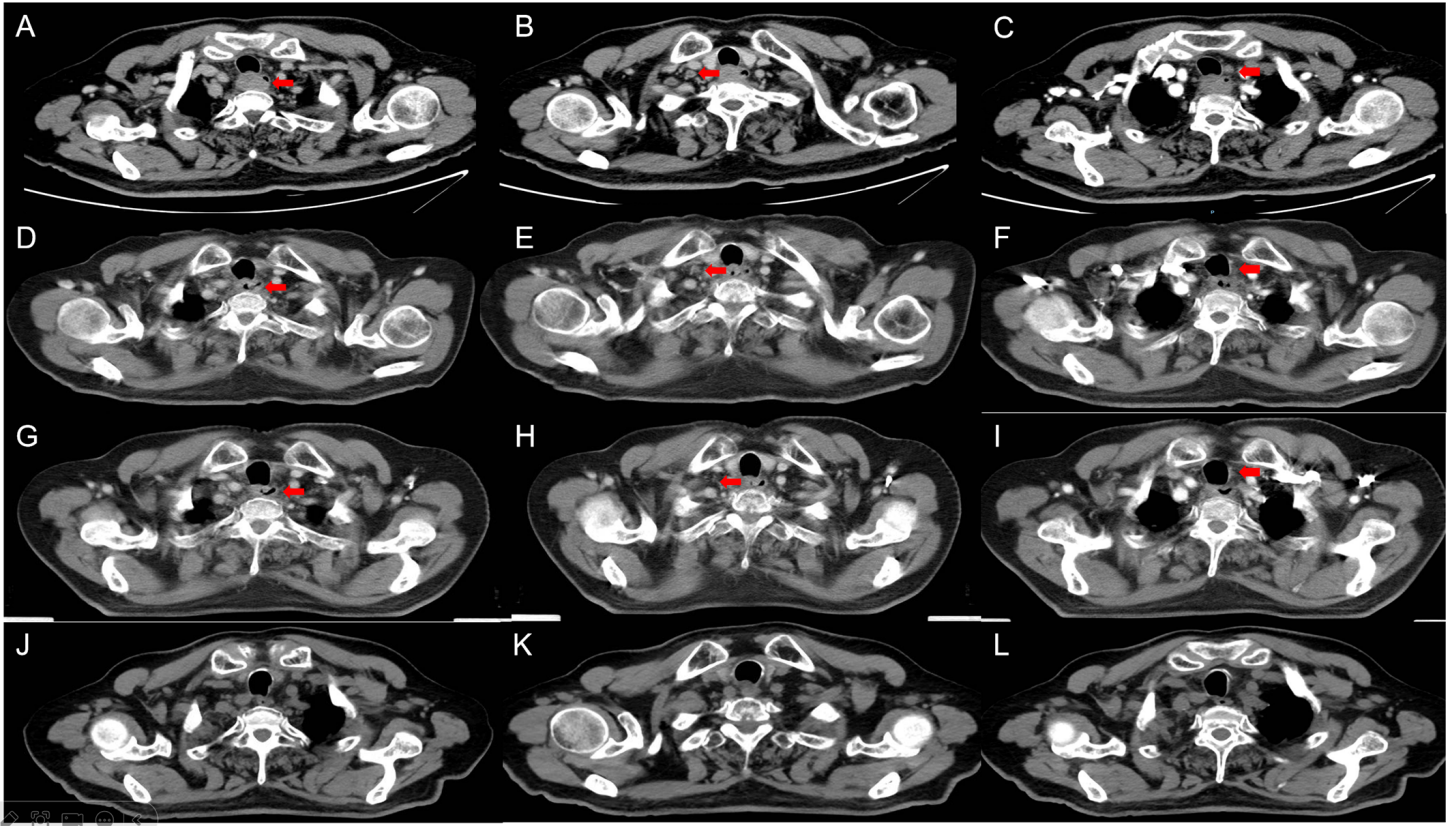
CT images during treatment. **(A–C)** CT images of the lesions at the initial treatment: esophageal lesions **(A)**, supraclavicular lymph node lesion **(B)**, and paratracheal lymph node lesion **(C)**. **(D–F)** CT images after nine treatment sessions. **(G–I)** CT images at the end of radiotherapy. **(J–L)** CT images from the follow-up examination on July 30, 2024.

**Figure 2:**
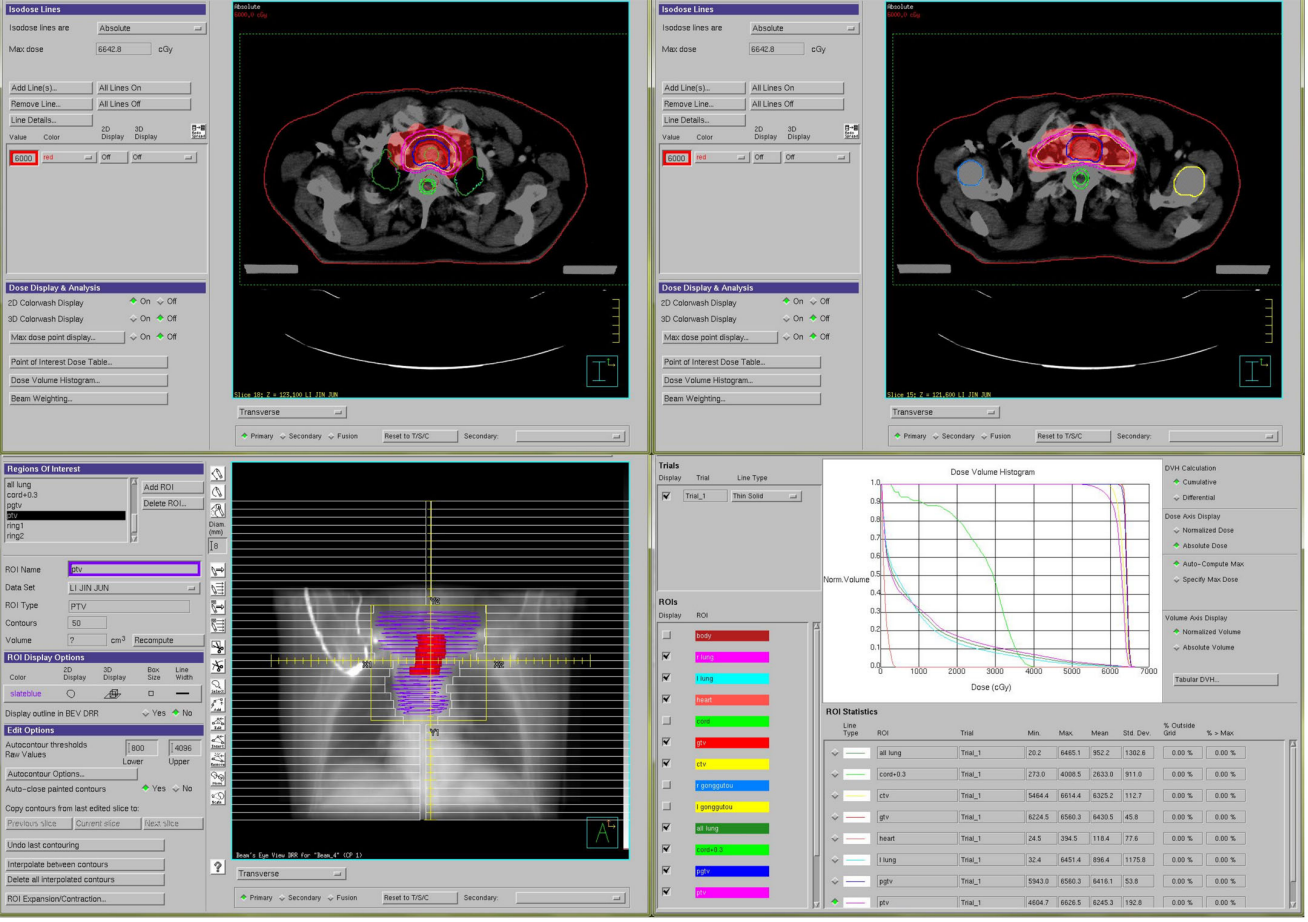
The intensity-modulated radiation therapy (IMRT) plan for this patient illustrates the precise delivery of radiation dose to the tumor and lymphatic drainage areas.

**Figure 3:**
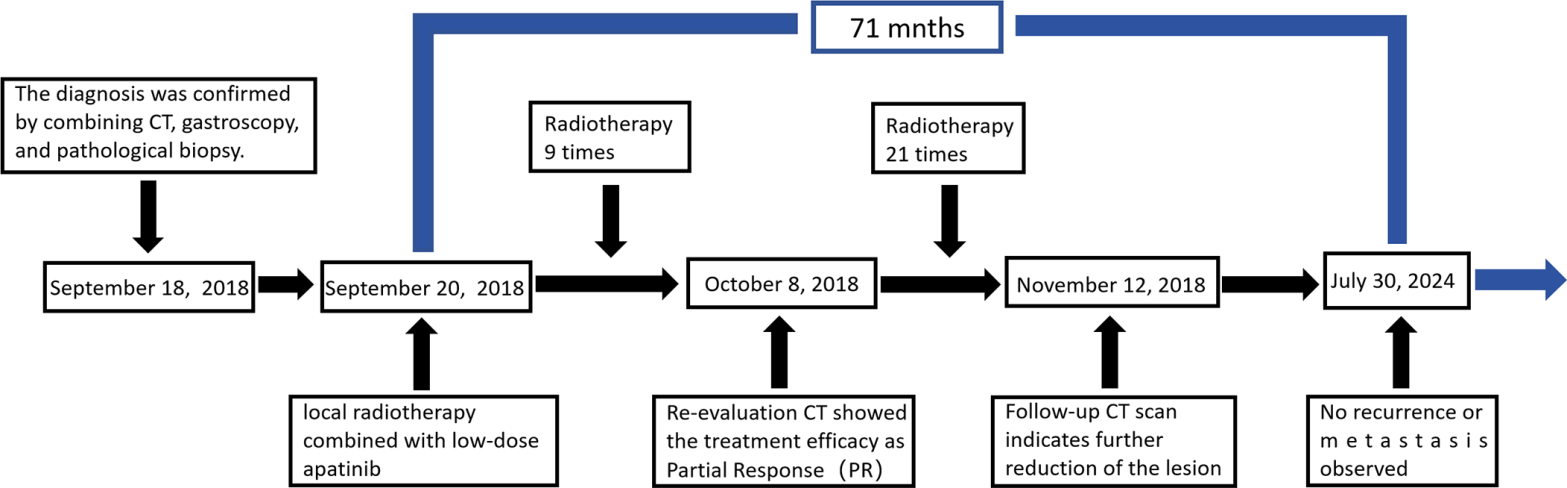
Timeline of the patient’s treatment.

In September 2018, an 81-year-old male presented with progressive dysphagia lasting more than 50 days. Throughout the course of the disease, he experienced no chest pain, nausea, vomiting, black stool, or hematemesis, but his symptoms progressively worsened. The patient sought treatment at our hospital after undergoing gastroscopy at a local hospital, suspecting EC. He was admitted for a physical examination with an Eastern Cooperative Oncology Group (ECOG) score of 0 and moderate nutritional status. No superficial lymph nodes were palpated, and no abnormalities were found during the heart, lung, and abdominal examinations. Contrast-enhanced chest computed tomography (CT) performed on September 10, 2018, revealed uneven thickening of the cervical to upper thoracic esophageal wall, with a maximum thickness of approximately 2.4 cm (**Figure 1A**), suggestive of cervical to upper thoracic esophageal carcinoma. The surrounding fat planes were poorly defined, with enlarged lymph nodes in the bilateral supraclavicular fossae (**Figure 1B**), paratracheal region (**Figure 1C**), aortic arch area, and tracheal bifurcation, indicating possible metastasis. Gastroscopy showed a medullary neoplasm protruding into the lumen approximately 20 cm from the incisors, exhibiting circumferential growth with luminal narrowing and deformity, preventing endoscopic passage. Five tissue samples were obtained from the stenotic areas for histological examination. Pathological results (September 15, 2018) confirmed the diagnosis of squamous cell carcinoma. The carcinoembryonic antigen level was 5.39 ng/mL, and fasting blood glucose was 7.20 mmol/L. The patient had a history of hypertension spanning over 10 years, with peak blood pressure readings of 150–160/120 mmHg. He was taking sustained release felodipine tablets (5 mg once daily) for blood pressure control, which was well managed. In 2010, he was diagnosed with type 2 diabetes mellitus and glycemic control was achieved with treatment. There was no family history of genetic diseases. Considering the patient’s symptoms, signs, and auxiliary examinations, he was diagnosed with cervical upper thoracic esophageal squamous cell carcinoma (cT3N2M0 Stage III) based on the 11th edition of the Japanese Esophageal Society esophageal TNM staging criteria. Given the patient’s advanced age, multiple comorbidities, and late-stage disease, a multidisciplinary discussion concluded that surgical intervention for cervical-to-upper thoracic EC would be highly challenging. Though radical chemoradiotherapy was recommended, the patient and their family declined it due to concerns about the toxic side effects of chemotherapy, which the patient cannot tolerate. Given the patient’s unresectable LA-ESCC and the inferior efficacy of radiotherapy alone compared to concurrent chemoradiotherapy, they opted to proceed with radiotherapy combined with the targeted therapy using apatinib. During treatment, patients should be advised to enhance their nutrition and avoid consuming very hot or coarse foods. The diet should primarily consist of liquid or semi-liquid options to minimize irritation to the esophageal mucosa and reduce the risk of tracheoesophageal fistula (TEF). Throughout the course of the disease, patients should closely monitor for symptoms such as cough while eating, black stools, cough, fever, and others. IMRT was employed, targeting the gross tumor volume (GTV), which included the esophageal wall thickening identified on CT and endoscopy, as well as lymph nodes with a short-axis diameter of ≥ 0.8 cm. The clinical target volume (CTV) extended 0.5–0.8 cm radially from the GTV and 2.5–3.0 cm longitudinally, encompassing the relevant lymphatic drainage areas. The planning target volume (PTV) was defined as a uniform 0.5 cm expansion around the CTV. Radiotherapy was delivered in daily fractions of 1.82 Gy over 6.5 weeks (12), achieving a total dose of 60 Gy to the PTV (**Figure 2**). The selection of the 60 Gy irradiation regimen was based on achieving superior conformality to the tumor target volume using IMRT technology (13). Moreover, involved-field irradiation helped protect patients’ normal tissues and reduced treatment side effects without compromising therapeutic efficacy (14). On September 20, 2018, the patient began local radiotherapy combined with low-dose apatinib (250 mg/day) as concurrent targeted therapy until the completion of radiotherapy. After nine sessions, the patient reported significant improvement in dysphagia. Given that rapid tumor regression may increase the risk of TEF, a follow-up contrast-enhanced chest CT on October 8, 2018, showed marked tumor reduction, including shrinkage of the supraclavicular and mediastinal lymph nodes. The walls of the esophagus and trachea remained intact, and no local ulcer formation was observed (**Figures 1D-F**). Radiotherapy was continued at the target dose, and a subsequent CT on November 12, 2018, revealed slight thickening of the esophageal wall without significant lymphadenopathy in the previously involved regions (**Figures 1G-I**). Throughout the treatment, the patient did not experience any severe adverse reactions (Grade III/IV). Specifically, after a 6-day follow-up blood test indicated a platelet count of 95 g/L, oral platelet-stimulating therapy was administered. The lowest platelet level during treatment reached 89×10^9/L and normalized one month after radiotherapy. A urinalysis performed 6 days after starting apatinib treatment revealed 0.3 g/L proteinuria with a glomerular filtration rate of 72 mL/min. A 24-hour urine protein quantification was normal, while blood pressure stabilized at 159/81 mmHg. The patient exhibited no bilateral lower limb edema or dizziness. After adding 0.15 g of irbesartan daily, weekly urinalysis confirmed negative proteinuria, with blood pressure fluctuating between 130–150 and 75–85 mmHg. During treatment, mild nail bed peripheral skin desquamation occurred without erythema, ulceration, sensory abnormalities, nausea, vomiting, chest pain, liver/kidney impairment, hematemesis, or melena. Follow-up contrast-enhanced chest CTs performed on February 27, 2019, September 16, 2020, September 6, 2022, and July 30, 2024, showed no significant thickening of the affected esophageal segment or enlargement of previously positive lymph nodes (**Figures 1J-L**). As of July 2024, the patient has had no tumor recurrence, and the treatment timeline is shown in **Figure 3**.

## 3 Discussion

The prognosis for patients with unresectable LA-ESCC is generally poor. Radiotherapy is the primary treatment modality for this patient population (4); however, its response and survival outcomes remain suboptimal and are accompanied by a high recurrence rate (15). This may be associated with radioresistance, in which hypoxia plays a significant role (16). Apatinib is an oral small-molecule tyrosine kinase inhibitor which works by suppressing tumor angiogenesis (17) and induces tumor vascular normalization (18), thereby improving tumor hypoxia and hypoperfusion. In addition, when combined with radiotherapy, apatinib inhibits DNA damage repair (19), reverses the immunosuppressive tumor microenvironment, and enhances tumor cell sensitivity to radiation (20).Similar findings from preclinical studies have been confirmed in numerous clinical trials. For example, a meta-analysis of patients with advanced pancreatic cancer demonstrated that apatinib when combined with radiotherapy improved the overall survival rate (11). Furthermore, in studies assessing the efficacy of apatinib combined with radiotherapy in non-small cell lung cancer patients with brain metastases showed that this combination therapy significantly reduced symptoms such as headaches and vomiting caused by the metastases and effectively delayed the progression of intracranial lesions compared to radiotherapy alone (21). In prostate cancer patients with bone metastases, the combination of apatinib and radiotherapy has proven to be more effective than targeted therapy alone in alleviating bone pain and altering prostate-specific antigen levels (22). Additionally, the combination of apatinib and radiotherapy did not increase the incidence of toxic reactions or side effects compared to radiotherapy administered individually (11, 21, 22). Although these clinical studies were limited by small sample sizes, they suggested that apatinib, when used alongside radiotherapy, can boost the therapeutic efficacy without compromising tolerable adverse reactions.

Patients with unresectable LA-ESCC may experience greater survival benefits from concurrent chemoradiotherapy (CCRT) compared to other treatment regimens (15). However, this approach also increases the risk of adverse effects (15), particularly in elderly patients whose physical status and tolerance often limit CCRT use (23). Numerous retrospective analyses have examined compliance with standard CCRT treatment in elderly patients, revealing that less than 40% of them can complete their scheduled treatment (9, 24). Japanese researchers have reported that simultaneous chemoradiotherapy for patients with EC aged > 80 years did not demonstrate a significant overall survival benefit over radiotherapy alone (25). Consequently, there is an urgent need to assess the efficacy of alternative and less toxic treatment options in elderly patients with unresectable LA-ESCC. A small-sample clinical study conducted by Li et al. demonstrated the therapeutic efficacy of apatinib monotherapy for unresectable esophageal squamous/adenocarcinoma (26). Hu et al. also confirmed that apatinib combined with concurrent chemoradiotherapy significantly improved the PFS of patients with LA-ESCC, enhanced the treatment response rate, and maintained manageable adverse reactions (27).In the present case, the patient was diagnosed with LA-ESCC, which was not amenable to surgical treatment. Given the patient’s advanced age of 82 years, underlying conditions such as hypertension and diabetes, and refusal of concurrent chemoradiotherapy, we decided to implement a radical treatment regimen combining apatinib with IMRT after thorough communication with the patient and their family. The patient achieved a PFS of up to 71 months. These results suggest that this treatment approach may have potential value for elderly patients with LA-ESCC who are ineligible for surgery.

In elderly patients, tumors often exhibit slower growth, characterized by increased extracellular matrix, reduced angiogenesis, and altered vessel morphology (28). As a result, EC in the elderly frequently displays relatively indolent biological behavior and tends to remain locally invasive. Previous studies found that radiotherapy or chemoradiotherapy combinations in patients with EC aged 80 years or older achieved a median survival of 42.5 months, while radiotherapy alone resulted in a 3-year PFS of 26.6%. However, most enrolled cases were stage I–III (25). In contrast, the present case involved supraclavicular lymph node metastasis, classified as T3N2M1 (stage IV) by the same staging criteria. Previous analyses comparing combined chemoradiotherapy to radiotherapy alone for ESCC showed a 5-year survival rate of 0% in the radiotherapy-only groups (29). These findings suggest that apatinib combined with radiotherapy may provide clinical benefits for elderly patients with unresectable LA-ESCC. Additionally, this case differs from previous clinical studies on the use of apatinib, either in combination with or without radiotherapy, for the treatment of advanced or recurrent ESCC in second-line and subsequent therapies. First, this study employed apatinib combined with radiotherapy alone, unlike prior monotherapy (26) or chemoradiotherapy combinations (27). Second, the patient in this case was over 80 years old and received apatinib at a dose of 250 mg/day. However, previous studies did not include patients over 80 years old, and the baseline dose of apatinib was 500 mg/day (26, 27). To the best of the authors’ knowledge, this is the first case of apatinib combined with radiotherapy for elderly patients with unresectable LA-ESCC.

Notably, a substantial proportion of TEF cases are secondary to EC (30), particularly in patients with T4-stage disease or pre-existing esophageal strictures (31). Previous studies have documented that the use of bevacizumab following chemoradiotherapy for lung cancer may contribute to TEF formation (32, 33). This underscores the need for heightened vigilance regarding such complications when combining apatinib with local radiotherapy in the management of unresectable LA-ESCC. In the present case, the patient had pre-existing esophageal strictures. When significant improvement in feeding obstruction was observed on the ninth day of treatment, we promptly performed a chest contrast CT scan to rule out fistula formation. Since this was only a single case, the evidence level is limited; the limitation lies in our inability to determine whether this treatment model is applicable to other elderly patients with LA-ESCC. Therefore, further research and clinical trials are necessary to validate the efficacy of this treatment strategy. For elderly patients with advanced local disease and multiple comorbidities, appropriate treatment decisions should be based on a comprehensive assessment of overall condition, expected survival, and treatment tolerance.

## Data Availability

The original contributions presented in the study are included in the article/Supplementary Material. Further inquiries can be directed to the corresponding author.
